# *Announcement*: World Arthritis Day 2017

**DOI:** 10.15585/mmwr.mm6640a5

**Published:** 2017-10-13

October 12, 2017 is World Arthritis Day (http://www.worldarthritisday.org). First observed in 1996, World Arthritis Day serves as a focus for organizations and individuals to work toward increasing awareness of arthritis and other rheumatic conditions worldwide. In the United States, 54 million adults (*1*) have some form of arthritis or other rheumatic condition. By 2040, the number of adults with arthritis is projected to increase 49% to 78.4 million, and the number of adults with arthritis-attributable activity limitation will increase 52%, from 23 million to 34.6 million (*2*).

The theme of this year’s World Arthritis Day is “Don’t delay. Connect today.” Early diagnosis and professionally guided management is important for maintaining a good quality of life, particularly for persons with inflammatory arthritis. There are four other things that persons with arthritis can do to help manage their arthritis: 1) learn arthritis management strategies, 2) be physically active, 3) maintain a healthy weight, and 4) protect their joints. Self-management education programs help persons with arthritis learn the strategies and gain the confidence to manage their condition, and improve pain control and function. Physical activity decreases pain, improves function, and delays disability; low impact, moderate intensity activities such as walking, cycling, water exercise, and fitness classes are safe and effective for persons with arthritis. Maintaining a healthy weight can limit disease progression and activity limitation, and avoiding joint injuries from sports or occupation can reduce the likelihood of developing osteoarthritis. Additional information on these interventions is available at https://www.cdc.gov/arthritis/interventions/index.htm.
